# Relationship Discovery and Hierarchical Embedding for Web Service Quality Prediction

**DOI:** 10.1155/2022/9240843

**Published:** 2022-10-05

**Authors:** Hualong Bu, Jing Xia, Qilin Wu, Liping Chen

**Affiliations:** ^1^School of Information Engineering, Chaohu University, Hefei 238000, China; ^2^School of Mathematics and Statistics, Chaohu University, Hefei 238000, China

## Abstract

Web Services Quality Prediction has become a popular research theme in Cloud Computing and the Internet of Things. Graph Convolutional Network (GCN)-based methods are more efficient by aggregating feature information from the local graph neighborhood. Despite the fact that these prior works have demonstrated better prediction performance, they are still challenged as follows: (1) first, the user-service bipartite graph is essentially a heterogeneous graph that contains four kinds of relationships. Previous GCN-based models have only focused on using some of these relationships. Therefore, how to fully mine and use the above relationships is critical to improving the prediction accuracy. (2) After the embedding is obtained from the GCNs, the commonly used similarity calculation methods for downstream prediction need to traverse the data one by one, which is time-consuming. To address these challenges, this work proposes a novel relationship discovery and hierarchical embedding method based on GCNs (named as RDHE), which designs a dual mechanism to represent services and users, respectively, designs a new community discovery method and a fast similarity calculation process, which can fully mine and utilize the relationships in the graph. The results of the experiment on the real data set show that this method greatly improved the accuracy of the web service quality prediction.

## 1. Introduction

More and more developers have paid attention to web services in the form of Web APIs [1]. By the end of March 22, 2022, ProgrammableWeb (the largest web service platform) has registered more than 24,417 APIs. Developers can access services through simple APIs, which provide a great convenience for development. As the number of services is increasing, users have only called a small number of services in reality, resulting in most service qualities being unknown in advance. Therefore, how to appropriately predict the quality of the web service becomes a serious issue [2].

Accompanied by the rising of deep learning technology, various deep web service quality prediction models have been continuously proposed [3–5]. Deep learning methods such as CNN and RNN can extract hidden information that cannot be treated by traditional matrix decomposition technology [6, 7] to improve the accuracy, but these methods do not take the topology information into account at all. In recent years, numerous graph convolutional networks (GCNs) have been proposed [8–12]. GCNs can integrate feature information from local graph neighborhood, which have been demonstrated to be powerful for graphical representation [13–15]. Neural Graph Collaborative Filtering (NGCF) [13] exploits the user-service graph structure and integrates the users and services interactions into the embedding process. PinSage [14] designs an efficient random walk method with graph convolutions to generate node embedding that incorporates both graph structure and node information. LDNM [15] transforms each service document into feature vectors by using LDA and Doc2vec, then applies Node2vec and MLP neural network for Web Service Classification.

Although GCNs have achieved great achievements in web service quality prediction, we argue that there are still suffering from the following challenges.

**Figure 1:**
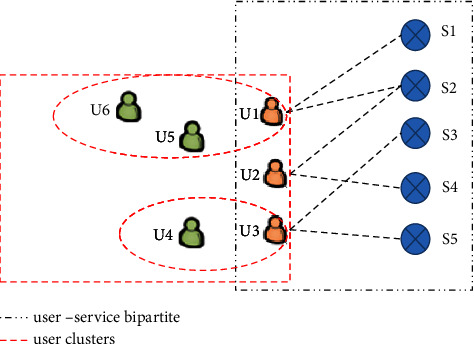
An illustration of the user and service relationships.

First, the user-service bipartite graph is essentially a heterogeneous graph. As shown in Figure 1, the bipartite user-service graph contains four types of relationships such as (1) user-user shared-invoking relationship; (2) user-user potential relationship; (3) user-server relationship; and (4) server-server relationship.

Taking the user node **u1** as an example, the first-order neighborhood of user **u1** contains the service nodes **s1** and **s2** which indicates the user-server relationship, and the second-order neighborhood contains the user node **u2** that has called the service **s2** before, which indicates the user-user shared-invoking relationship.

Taking the service node **s1** as an example, the first-order neighborhood of **s1** contains the user nodes **s1**, and the second-order neighborhood contains the service node **s2**. Node **s1** and node **s2** form the server-server relationship.

Furthermore, taking user **u1**, user **u5**, and user **u6** as an example, these users share characteristics (e.g., they may have common interests), but they have not revoked the same service in the past. In this way, users **u1**, **u5**, and **u6** form a user-user potential relationship with each other. Generally speaking, without prior domain knowledge, the user-user potential relationships are unknown in advance.

Previous GCN-based models have only focused on using one or more of these relationships. Therefore, how to fully mine the above relationships and make use of these relationships is critical to improve the prediction accuracy.

Second, when aggregating node features with the help of topology information, traditional methods must calculate the similarity. The existing similarity calculation research mainly focuses on how to improve the accuracy of downstream tasks, and it is necessary to traverse all samples one by one during traversal [16, 17]. Obviously, similarity calculation is a very time-consuming process when the number of samples is large. In order to reduce the computational complexity, a more efficient similarity calculation method needs to be adopted.

In addition, we notice that the web service quality prediction methods based on GCN have also faced the matrix sparsity problem to be solved. To address the aforementioned challenges simultaneously, this article proposes a novel relationship discovery and hierarchical embedding method (RDHE) based on GCNs for web service quality prediction, which can fully utilize four kinds of relationships to achieve better prediction accuracy. Given a user-service bipartite graph or QoS matrix, we first reconstruct the user-neighborhood graph and the service-neighborhood graph, respectively. Second, we propagate and aggregate the features of nodes from their local neighborhood by user embedding module and service embedding module, respectively. Third, we cluster the user embedding and service embedding with the help of clustering component. In addition, we conduct the communities using the community discovery module to instruct the downstream clustering. After these, we fuse the user-based and server-based predictions for the ultimate prediction. The experimental results show that our RDHE method can greatly improve the accuracy. The main contributions of this work are summarized as follows.

1. A dual model to effectively learn neighborhood embedding from the user perspective and service perspective independently, which can lighten the damage caused by sparsity.
2. Two community discovery methods are proposed to instruct the user- and service-based predictions to improve the accuracy of the final prediction, which can fully mine and use the above four kinds of relationship.
3. A fast similarity calculation method to handle large-scale data, which can avoid the curse of dimensionality for downstream clustering tasks.
4. A large number of experiments are carried out on the real WS-Dream data set. The experimental results show that compared with the classic methods, the prediction precision of our RDHE method improves a lot.

The remainder of this article is organized as follows: Section 2 introduces the related work for Web Service Quality prediction.  Section 3 shows the whole framework.  Section 4 explains in detail the implementation process of the RDHE algorithm.  Section 5 presents the experimental results and analysis. We summarize the full text in the Conclusion section.

## 2. Related Work

### 2.1. Deep Learning Models for Embedding

With the development of cloud computing and the Internet of things, various web services are registered and invoked on the network, and the prediction of web service quality has become a popular research direction. Our work builds on some recent advances in graph neural network representation learning methods.

The concept of neural networks for graph data was first outlined by Gori et al. [18] and further elaborated by Scarselli et al. [19]. However, these initial methods required running expensive neural “message passing” algorithms to converge and were constrained by the scale of graph data, which was further improved by gated graph sequential neural networks [20] to graphs with <10,000 nodes, but the cost is still high.

A large number of approaches to “graph convolution” or graph convolutional networks (GCNs) have recently emerged, originating from the work of Bruna et al., which developed a graph convolution based on spectral graph theory [21]. Since then, many authors have proposed improvements, extensions, and approximations to these spectral convolutions [8–12, 22, 23], and achieved good results on tasks such as node classification and link prediction. These methods have consistently outperformed matrix factorization or random walk-based techniques (e.g., node2vec [24] and DeepWalk [25]), and their success has led to a surge of interest in applying GCN-based methods to applications such as recommender systems, of which Hamilton et al. [26] and Bronstein et al. [27] conducted a comprehensive survey of recent progress.

However, despite the success of GCN algorithms, the above works are mainly based on homogeneous graphs, ignoring the heterogeneous structure effects of graphs. Since the representation learning of heterogeneous graph neural network has the strong expressive ability and the characteristics of effectively combining node attribute features and structural information, it can not only solve the problems of network data such as data sparse [28], but also has achieved remarkable results in various downstream tasks, such as node classification [29], link prediction [30], node clustering [31, 32], and recommendation [33–35].

We found that the heterogeneity of the graph structure causes different node types to have different semantic features, and even if the node types are the same, there may be different community attributes (e.g., although they are all user nodes, their implied points of interest are different). Inspired by the community discovery algorithm [36], our work introduces community discovery (further discovering distinct communities of cohorts to guide downstream work) into graph neural network representation learning.

### 2.2. Similarity Calculation

Our method is also relevant to the field of similarity calculation. Similarity calculation indicators can evaluate the similarity between data, and provide an accurate and effective basis for data analysis. Previous studies on similarity calculation mainly focus on the following aspects:

1. The design of basic measurement indicators, which can be divided into distance-based measurement methods and similarity coefficient-based measurement methods [37, 38]. Distance-based measurement methods mainly include Euclidean distance, Manhattan distance, etc., while similarity coefficient-based measurement methods mainly include Cosine similarity, Pearson correlation coefficient, etc. Among them, the most common methods are the application of Euclidean distance and Cosine similarity.
2. Embedding similarity strategy design, this type of method mainly studies how to improve the guidance of similarity calculation for downstream tasks [16], such as ontology-based methods, semantic-based methods, and some other hybrid methods [17].

The above methods mainly consider the effectiveness or learning accuracy of the similarity measurement method itself, but due to the large amount of current data, the time performance of similarity calculation is insufficient. To this end, this article proposes a similarity calculation method based on a neural network. The main idea is to reduce the time required for similarity calculation as much as possible under the premise of ensuring accuracy.

## 3. The Whole Framework

**Figure 2:**
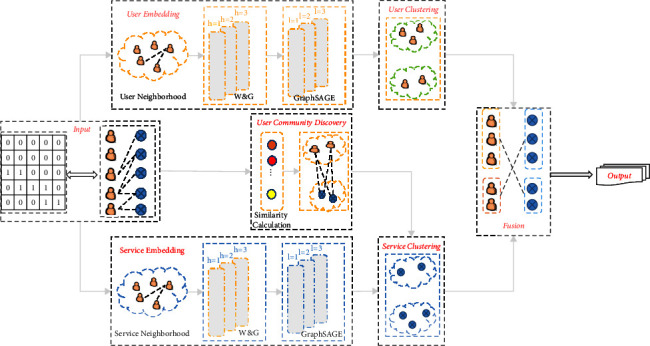
The framework of web service quality prediction. The input can be QoS matrix or corresponding bipartite graph. The output is the prediction of missing QoS values.

We will briefly describe the framework of our proposed method RDHE, as shown in Figure 2. The entire framework consists of six components:

- User embedding module, which contains the reconstruction of the User Neighborhood component, Wide and Deep Embedding component, and GraphSAGE Embedding components.
- Service embedding module, which contains similar components as user embedding module.
- User-community discovery module, in which a new community discovery method is proposed to instruct the service-based clustering.
- User clustering module, in which a clustering method is proposed for user clustering.
- Service clustering module, in which we get the service cluster and the user-cluster affiliation degree matrix.
- At last, a fusion operation is used to fuse the user-based prediction and server-based prediction.

## 4. The Proposed Prediction Method

### 4.1. Reconstruction of Neighborhood Graph

Given a QoS matrix *Q*^*m*×*n*^, *m* and *n* are the number of users and services, respectively. *X*^(*m*+*n*)×*d*^ is the feature matrix and *d* is the dimension of features. The corresponding bipartite graph *G*(*U*, *S*, *E*, *X*) can be simply derived from *Q*^*m*×*n*^. The bipartite graph *G*(*U*, *S*, *E*, *X*) is a typical heterogeneous graph, the direct neighbors of users are the service nodes and the direct neighbors of services are the user nodes.

Assume that it is more efficient to learn different neighborhood embedding from the user perspective and service perspective independently, rather than recursively updating neighborhood embedding regardless of the node types. Based on this, we reconstruct a user-neighborhood graph and a service-neighborhood graph from the *G*(*U*, *S*, *E*, *X*) or its corresponding QoS matrix directly. If user *u*_1_ and user *u*_2_ are connected to the same service, they become direct neighbors in the user-neighborhood graph. In other words, if the elements of the *i*-th column in the QoS matrix are nonzero, then corresponding users of service *i* become direct neighbors in the user-neighborhood graph. Likewise, if two services connect to the same user (the corresponding row's elements are nonzero), they become direct neighbors in the service-neighborhood graph. In this way, we can reconstruct the user-neighborhood graph *G*(*U*, *E*′,  *X*_*u*_),  *X*_*u*_ ∈ *ℝ*^*m*×*d*^ and service-neighborhood graph *G*′(*S*,  *E*^″^, *X*_*s*_),  *X*_*s*_ ∈ *ℝ*^*n*×*d*^.

It should be noted that *G*(*U*, *E*′, *X*_*u*_) contains two kinds of user relationship information, one is the user-user shared-invoking relationship which is constructed by the above method and is reflected in the topological structure of *G*(*U*, *E*′, *X*_*s*_); the other is the user-user potential relationship which contains the latent community-sharing information, and we will discover it through the community discovery module (more details will be discussed in Section 4.3).

### 4.2. Sparsity and Large-Scale Handling

In general, the feature matrixes of *X*_*u*_ and *X*_*s*_ are sparse and high-rank, which will lead to the failure of methods which directly based on the feature matrixes. Here, we adopt the Wide & Deep [39] learning model to better represent sparse features by simultaneously training a linear LDA model and a neural network model.

Given a user-neighborhood graph *G*(*U*, *E*′, *X*_*u*_) and its nodes feature matrix X_*u*_, we can get the representation as,

(1) $$\begin{array}{c} E_{u}^{w}=WD_{u}X_{u}\text{,} \end{array}$$

where *WD*_*u*_(∙) is a Wide & Deep learning module for users. Similarly, the service representation can be obtained by the Wide & Deep learning module as

(2) $$\begin{array}{c} E_{s}^{w}=WD_{s}X_{s}\text{.} \end{array}$$

We then output *E*_*u*_^*w*^and *E*_*s*_^*w*^ as inputs of GraphSAGE [40], which is a general framework that takes advantage of the node feature to efficiently embed large-scale data, and can generate embedding by sampling and aggregating features from a node's neighborhood as follows:

(3) $$\begin{array}{c} H_{N_{u}}^{t}={aggregate}_{u}^{t}\left(\left\{H_{i}^{t-1},i\in N_{u}\right\}\right)\text{,}\\ H_{u}^{t}=\sigma \left(W_{u}^{t}\;∙\;concat\left(H_{u}^{t-1},H_{N_{u}}^{t}\right)\right)\text{,}\\ H_{N_{s}}^{t}={aggregate}_{s}^{t}\left(\left\{H_{j}^{t-1},j\in N_{s}\right\}\right)\text{,}\\ H_{s}^{t}=\sigma \left(W_{s}^{t}\;∙\;concat\left(H_{s}^{t-1},H_{N_{s}}^{t}\right)\right)\text{,} \end{array}$$

where aggregate_*u*_^*t*^(∙) and aggregate_*s*_^*t*^(∙) are aggregating functions, *σ* is the activate function, *W*_*u*_^*t*^ and *W*_*s*_^*t*^ are the parameter matrixes which can be trained. *H*_*s*_^0^=*E*_*s*_^*w*^ and *H*_*u*_^0^=*E*_*u*_^*w*^ as original inputs.

Note that concat(∙) is referring to the layer-aggregation mechanism. Since concatenation does not require additional parameters to learn, it has been shown quite simply and effectively for graph neural networks. In addition to concatenation, other strategies such as max pooling, can also be applied. Here, we adapt the concatenation for its simplicity.

### 4.3. Clustering with the Instruction of Community Discovery

#### 4.3.1. Fast Similarity Calculation Process

Clustering divides a data set into different clusters according to a certain distance criterion so that the similarity of samples in the same cluster is as large as possible, and the difference of samples not in the same cluster is also as large as possible.

**Figure 3:**
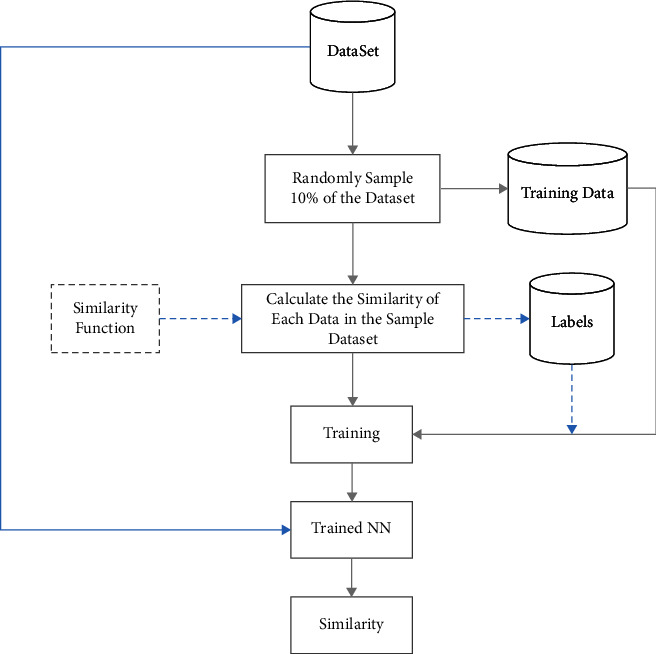
Fast similarity calculation.

Traditionally, a clustering algorithm usually only needs a similarity calculation function sim(*s*_*i*_, *s*_*j*_) to get started. Traditional similar calculation process needs to traverse the embedding one by one when calculating sim(*s*_*i*_, *s*_*j*_), which are suitable for small-scale data sets, but the operating time will increase exponentially with the expansion of the dataset size [38]. In this work, a neural network-based similarity calculation process is designed for downstream similarity calculation and reduced the time complexity as shown in Figure 3.

In Figure 3, in order to improve the similarity calculation performance, a network model that can fit the similarity mapping relationship of the dataset is established. BP neural network has excellent nonlinear approximation, self-learning, self-adaptation, and generalization ability. According to the universal approximation theorem, if a feedforward neural network has a linear output layer and at least one hidden layer with an activation function, as long as there are enough hidden neurons, it can approximate any continuous function in a finite-dimensional space with arbitrary precision. In actual use, the computational time and space overhead must be considered, the exact representation is usually abandoned, and the nonlinear mapping relationship between the dataset and the label set is approximated by finding suitable parameters on the basis of the approximate representation. This provides a theoretical basis for using BP neural network for fast similarity calculation.

After the similarity calculation formula is selected, the numerical value of the data and the similarity between the data can constitute a definite multi-input and multi-output nonlinear mapping relationship, so the BP neural network can be used to fit this mapping relationship. In order to reduce the time of traversal calculation, the model is trained based on the similarity of some samples, and the accuracy is exchanged for speed, and the nonlinear similarity mapping relationship of the complete data set is represented within the allowable error range, so as to calculate the approximate similarity of the complete data set.

We give a brief complexity analysis here. Set *X* ∈ *R*^*n*×*m*^. For tradition calculating method, the total time complexity is *O*(*mn*^2^). The time complexity of the neural network method can be divided into precise calculation part and network part. In the precise calculation part, let *b* be the sampling ratio, 0 < *b* < 0.5, the precise calculation complexity is *O*(*b*^2^*mn*^2^). Assuming the number of the inputted neurons per hidden layer is *m*, the hidden layer number is *t*, and the number of output neurons is 1, the time complexity of the neural network method is *O*((1 − b)*mnt*). Generally, *t* ≪ *n*, so the neural network method is more efficient than the traversal calculation method.

#### 4.3.2. User Clustering

We note the cluster center vector as the mean vector of the feature vectors of all the users in the cluster, and calculate the similarity between the feature vector of the user *u* in cluster *i* and the rating vector of cluster *i* as the user *u*'s interest in the cluster *i*.

The similarity calculation function is the widely used Pearson Correlation Coefficient (PCC). We use PCC to measure the similarities between vector *r*_*i*_ and *r*_*i*_, and get the user clusters *UC* on the user embedding *H*_*u*_^*t*^. We use the function *f*(*x*)=(*x*+1)/2 to project the PCC into range [0, 1]. The PCC is defined as:

(4) $$\begin{array}{c} sim\left(i,j\right)=\frac{\sum_{p\in P}\left(x_{ip}-{\bar{x}}_{i}\right)\left(x_{jp}-{\bar{x}}_{j}\right)}{\sqrt{\sum_{p\in P}{\left(x_{ip}-{\bar{x}}_{i}\right)}^{2}{\left(x_{jp}-{\bar{x}}_{j}\right)}^{2}}}\text{,} \end{array}$$

where *P* is the interaction of the two vectors.

Based on the assumption that users with more social relationships are more representative, we can derive the weighted cluster center vectors.

(5) $$\begin{array}{c} {WC}_{i}=\frac{\sum_{g\in UC\left(i\right)}\left|{relation}_{i}\left(g\right)\right|r_{u_{g}}}{\sum_{g\in UC\left(i\right)}\left|UC\left(i\right)\right|}\text{,} \end{array}$$

where *C*(*i*) represents the set of users in cluster *i*, relation_*i*_(*g*) represents the set of users who have a direct relationship with user *u*_*g*_ in cluster *i*.*r*_*u*_*g*__ is the feature vector of user *u*_*g*_.

Finally, we can obtain the user clusters *UC* and the rating matrix *UCR* by formula (4) and (5).

#### 4.3.3. Service Clustering with the Instruction of Community Discovery

Finding and utilizing community information on the original input QoS matrix without prior knowledge can be connected to the concept of community discovery. A fuzzy clustering algorithm based on interest preference is proposed here. The algorithm uses the user's behavior records and the clusters to which the services belong to find a set of users similar to the target user's interest preference.

Given the original input QoS matrix *Q*^*m*×*n*^, the users' ratings on services form a *m* × *n* rating matrix *R*=[*r*_*ij*_], where *r*_*ij*_ is the rating of user *u*_*i*_ on service *s*_*i*_, *r*_*i*_ is the rating vector of user *u*_*i*_. We note the community rating vector as the mean vector of the rating vectors of all the users in the community.

First, we use the PCC to calculate the similarity of the service embedding *H*_*s*_^*t*^ to get the service clusters *SC*.

Next, we derive the distribution of clusters *SC* which have been rated by *u*_*i*_ can be described as:

(6) $$\begin{array}{c} Dist\left(i\right)=\left(\frac{\left|S_{i}^{{SC}_{1}}\right|}{\left|S_{i}\right|},\frac{\left|S_{i}^{{SC}_{2}}\right|}{\left.{\left|S\right.}_{i}\right|},\ldots ,\frac{\left|S_{i}^{{SC}_{p}}\right|}{\left|S_{i}\right|}\right)\text{,} \end{array}$$

where *S*_*i*_represents the set of services rated by user *u*_*i*_, and *S*_*i*_^*SC*_*k*_^ represents the set of services rated by user *u*_*i*_in cluster *C*_*k*_.

Next, due to the sparse data of the user-service rating matrix, the user category preference vector also has a sparseness problem, we define a more effective similarity function than traditional Minkowski distance as follows:

(7) $$\begin{array}{c} sim\left(Dist\left(i\right),g_{j}\right)=\frac{1}{\tau_{i}}\overset{p}{\underset{k=1}{\sum }}\tau_{ik}\left({Dist\left(i\right)}_{k},g_{j,k}\right)\text{,} \end{array}$$

where $\tau_{ik}=\left\{\begin{array}{c} 1,{Dist\left(i\right)}_{k}\neq 0\\ 0,{Dist\left(i\right)}_{k}=0 \end{array}\right.$, *τ*_*i*_=∑_*k*=1_^*p*^*τ*_*ik*_. *l* is the number of clusters, *g*_*j*_ represents a certain cluster center vector, *j*=1,2,…, *l*. *g*_*j*,*k*_ is the *k*-th element in *g*_*j*_.

Then we derive the objective function:

(8) $$\begin{array}{c} Objective\left(USA,G\right)=\overset{m}{\underset{i=1}{\sum }}\overset{l}{\underset{j=1}{\sum }}u_{ij}^{2}sim\left(Dist\left(i\right),g_{j}\right)\text{,} \end{array}$$

where *u*_*ij*_ ∈ [0,1] represents the affiliation degree of user *u*_*i*_ to cluster *C*_*j*_ and ∑_*k*=1_^*l*^*u*_*ij*_=1. *USA*=*u*_*ij*_^*m*×*l*^ represents the user-cluster affiliation degree matrix. *G*=( *g*_1_, *g*_2_,…, *g*_*l*_)^*T*^ represents the center cluster matrix.

Finally, we update the values of the user-cluster affiliation matrix *USA* and cluster center matrix *G* through iterative operation, and gradually reduce the error of the objective function to the pre-set convergence threshold and terminate the iteration.

### 4.4. Joint Prediction

In order to reduce the influence of the redundant neighbors on the prediction, we predict the missing QoS values *P*_*u*_(*i*, *j*) of user *j* as follows:

(9) $$\begin{array}{c} P_{u}\left(i,j\right)=\frac{\sum_{i=1}^{top-k}\left({qos}_{u_{i}}\times {UCR}_{it}\right)}{top-k}\text{,} \end{array}$$

where *u*_*i*_ is the *i*-th user in the cluster *UC*_*t*_ which user *u*_*j*_ belongs to, *qos*_*u*_*i*__ represents the QoS value of user *u*_*i*_, *UCR*_*it*_ means the rating score of user *u*_*i*_ to cluster *UC*_*t*_, and top − *k* represents the number of how many neighbors to be selected.

The formula for predicting missing values *P*_*s*_(*i*, *j*) of service *j* based on the service clustering result is as follows:

(10) $$\begin{array}{c} P_{s}\left(i,j\right)=\frac{\sum_{i=1}^{top-k}\left({qos}_{u_{i}}\times {US}_{it}\right)}{top-k}\text{,} \end{array}$$

where *u*_*i*_ is the *i*-th affiliated user of the server cluster *SC*_*t*_ which user *u*_*j*_ affiliated to, *qos*_*u*_*i*__ represents the QoS value of user *u*_*i*_, *US*_*it*_ means the affiliation degree of user *u*_*i*_ to cluster *SC*_*t*_, and top − *k* represents the number of how many neighbors to be selected.

To fully consider the similarity of similar users and similar services, this article combines formulas (9) and (10) to propose a hybrid prediction method as follows:

(11) $$\begin{array}{c} P_{u,s}\left(i,j\right)=\lambda \times P_{u}\left(i,j\right)+\left(1-\lambda \right)\times P_{s}\left(i,j\right)\text{.} \end{array}$$

The parameter *λ* ∈ [0,1] indicates that the predicted value depends on the proportion of similar users and similar services. When *λ* = 1, only the user-based method is used for prediction; when *λ* = 0, only the service-based method is used for prediction. If user *u* does not call any service that has been used or service *s* has not been called by any user, the average of all service QoS values will be used for prediction.

### 4.5. Model Training

To optimize the model parameters of RDHE, we need to optimize an objective function. In the training process, we denote S^+^ as positive samples and denote S^−^ as negative samples, respectively. Given user *u*,*y*_*u*,*s*_ is the ground truth label, if user *u* has invoked the service *s*, then *y*_*u*,*s*_=1, otherwise *y*_*u*,*s*_=0, ${\hat{y}}_{u,s}$ is the predicted label for service *s*. The loss function is derived as:

(12) $$\begin{array}{c} -\underset{u}{\sum }\underset{i\in s^{+}\cup s^{-}}{\sum }\left\{y_{u,s}\log\;P\left({\hat{y}}_{u,s}=1\right)+\left(1-y_{u,s}\right)\log\;P\left({\hat{y}}_{u,s}=0\right)\right\}\text{.} \end{array}$$

Our model contains service embedding *E*_*s*_^*F*^ and user embedding *E*_*u*_^*F*^, they are hierarchical trained and learned together. Since the raw features are very large and highly sparse for large-scale graphs, we do not use one-hot vectors to represent the nodes. By embedding high-dimensional sparse features into a low-dimensional latent space, the model can be easy to train. It is worth noting that, in terms of model size, the majority of the RDHE model parameters come from the embedding. We adopted the Cluster-GCN Mini-batch Trick [41] and Adam [42] optimization for GCNs.

Overfitting is another long-term problem for optimizing deep neural network models. The dropout strategy [43] is a common method to solve the overfitting of the neural network, which has the characteristics of simplicity and good effect. Here, we employ node and feature dropout strategies to prevent RDHE from overfitting. When the parameters, only part of them will be updated. Moreover, as a dropout is disabled during testing, the whole network is used for prediction.

## 5. Experiment

In this work, we performed a lot of experiments on real-world datasets to evaluate our RDHE method. We hope to answer the following questions:

- How does RDHE perform compared to the state-of-the-art methods?
- How do different hyper-parameter settings affect RDHE?

### 5.1. Experimental Data and Evaluation Indicators

In our experiments, in order to evaluate the effectiveness of the prediction method proposed in this article, we use the WS-Dream dataset [44]. The two matrices in this dataset contain 1974675 response time (RT) and throughput (TP) data generated by 339 users calling 5825 services. There is a QoS record generated by invoking between each user and each Web service. The real recorded values of RT are between [0, 1].

To evaluate the effectiveness of service quality prediction, this work uses the mean absolute error (MAE) and the root mean square error (RMSE) indicators for test, which are defined as follows.

(13) $$\begin{array}{c} MAE=\frac{\sum_{u,s}\left|r\left(u,s\right)-p\left(u,s\right)\right|}{N}\text{,}\\ RMSE=\sqrt{\frac{\sum_{u,s}{\left(r\left(u,s\right)-p\left(u,s\right)\right)}^{2}}{N}}\text{,} \end{array}$$

where *r*(*u*, *s*) and *p*(*u*, *s*) represent the actual QoS and predicted QoS values, respectively, and *N* represents the number of predictions. It can be seen from the formula that the smaller the values of MAE and RMSE, the better the prediction performance, and the RMSE is more sensitive to the prediction error.

### 5.2. Baselines

We compare our proposed RDHE method with 10 state-of-the-art methods, which is described as follows:

1. LDA [45]: LDA is an unsupervised topic modeling algorithm, which has been widely used in recommender systems. Here, each service is associated with a distribution on the topic of users, each user group is a distribution of users, and we use LDA to discover hidden relationships between users and services in the prediction task.
2. Node2Vec [24]: Node2Vec is an improved version of DeepWalk that takes into account structure and homogeneity. In this method, we use the Node2vec model to maintain node neighbor information from the user- and service-neighborhood graphs for downstream tasks.
3. GCN [8]: GCN is a classic semi-supervised model for extracting graph features. In this article, we treat both the services and users as nodes, then use the GCN to obtain the embedded representation of the graph.
4. Wide & Deep [39]: in this method, we only use the Wide & Deep method to obtain user and service embedding for downstream similarity calculation and prediction tasks.
5. GraphSAGE [40]: in this method, we only use the GraphSAGE module to obtain user and service feature representations for downstream tasks.
6. NGCF [13]: in this method, we use the NGCF module to exploit the user-service graph structure by propagating high-order connectivity embedding on it; next the prediction task is imposed on it.
7. PinSage [14]: in this method, we use a PinSage module to combine to generate embedding of nodes that incorporate both graph structure as well as node feature information. Then we perform similarity calculation and prediction tasks on it.
8. KGAT [34]: in this method, we use a KGAT module to propagate the embedding by aggregating information from the constructed knowledge graph for prediction.
9. GraphRec [12]: in this method, we use a GraphRec module to capture interactions and heterogeneous strengths in the user-service graph for prediction.
10. NIA-GCN [35]: in this method, we adopt the NIA-GCN module to capture relationships between pairs of neighbors at each GCN layer for prediction.

LDNM is a fusion method of LDA, Doc2vec, and Node2vec, which have been adopted, respectively, thus we will not compare it.

### 5.3. Parameter Setting

We implement our method on the basis of Pytorch2 and use the Adam optimizer for all neural network-based methods. All the baselines and our algorithm are fully trained with up to 500 epochs, and the number of negative samples is 20. Moreover, we use validation set data for parameter tuning for all the methods.

In the real world, most of the users have only called a few services, therefore, in this work, to make the experiments more realistic, we randomly delete a certain number of QoS values from the initial RT and TP matrices to generate low-density matrices. For example, a matrix density of 5% means that we randomly select 5% of the QoS values to predict the remaining 95% of the QoS values. The removed QoS values are used as expected values to study prediction accuracy. Each training set data contain 10% of the validation set data; for example, 50% of the training set size means that 40% of the data are used for training and the remaining 10% of data are for validation.

The default values of the parameters and hyper-parameters of the baselines are shown in Table 1. If there are multiple datasets and the parameters are not specified, then the default parameters are the same. Specifically, we set the number of topics to 20 for all LDA-contained methods, where each latent topic corresponds to a specific service cluster. For our method, the Wide & Deep and GraphSAGE parameters are the same as in Table 1. We set the hidden layer the same as the embedding size and the activation function as ReLU. We used four hidden layers for the NN components of the Similarity Calculation and Prediction Module and its learning rate = 0.03, dropout = 0.5, and optimizable parameter *δ*=0.6. The effect of different values of *λ*, top-*k*, and other hyper-parameters will be shown in chapter 5.4.

### 5.4. Experiment Result

We first compare the prediction accuracy of different methods and then compare the effect of different parameters in RDHE, all the results and details will be discussed below.

#### 5.4.1. Prediction Accuracy Comparison

In the experiment, the matrix density is set to 0.1–0.3 on the WS-Dream dataset (divided into RT and TP sub-datasets), and gradually increases at an interval of 0.05. We set *k*=3 and *λ*=0.4 to obtain the MAE and RMSE results. The experimental results are compared in Tables 2 and 3.

Tables 2 and 3 compare the prediction accuracy of 10 state-of-the-art methods with RDHD. It can be seen that our RDHE method has obtained better experimental results with both MAE and RMSE indicators, compared to all other baseline methods. Take the result of the MAE indicator as an example, the RDHE method is better than the four classic graph algorithms (Node2vec, GCN, Wide & Deep, and GraphSAGE) with 18.84%, 21.50%, 15.47%, and 12.50% achievements, respectively; it also achieves 11.11%, 9.19%, 9.19%, 5.62%, and 3.45% improvement than NGCF, PinSage, KGAT, GraphRec, and NIA-GCN, respectively. Similarly, take the result of the RMSE indicator in Table 3 as an example, the RDHE method achieved 14.48%, 16.85%, 14.19%, 13.74%, 12.33%, 8.51%, 7.98%, 11.21%, and 8.04% accuracy improvement than the nine GCNs methods based on the RMSE indicators.

Tables 2 and 3 also show the prediction accuracy changes of the different methods as the matrix density increases. As the matrix density increases, each method achieves better results than at lower densities, and the RDHE method improves even more.

#### 5.4.2. The Influence of *λ* for Prediction Accuracy

**Figure 4:**
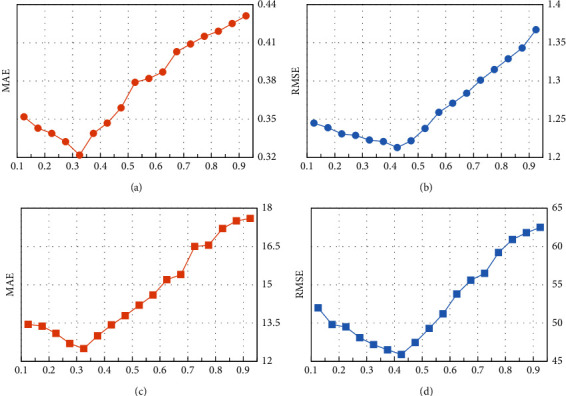
The influence of parameter *λ*. (a) RT attribute. (b) RT attribute. (c) TP attribute. (d) TP attribute.

As shown in (11), the parameter *λ* is a weight parameter, which determines the degree of dependence of the results based on the user's similarity and service's similarity. For example, if *λ* = 1, only the user-based method is used for prediction, and if *λ* = 0, only the service-based method is used for prediction. In order to study the influence of *λ* on the prediction results, the matrix density is set to 0.1, the number of similar users and the number of similar services is 6, and the *λ* ranges from 0.1 to 0.9 with an interval of 0.05. The experimental results are shown in Figure 4.

Among them, Figures 4(a) and 4(b) are the experimental results of the response time attribute, and Figures 4(c) and 4(d) are the experimental results of the throughput attribute. The experimental results in Figure 4 show that *λ* is one of the decisive factors for RDHE prediction accuracy. As shown in Figures 4(a) and 4(c), as the value of *λ* increases, the prediction accuracy reaches the maximum value when *λ* = 0.3. Meanwhile, as shown in Figures 4(b) and 4(d), the prediction accuracy reaches the maximum value when *λ* = 0.4. This shows that a suitable *λ* value can achieve better prediction accuracy. At the same time, the results in Figure 4 show that when the datasets are different, the optimal value of *λ* is also different.

#### 5.4.3. The Influence of Top-*k* for Prediction Accuracy

**Figure 5:**
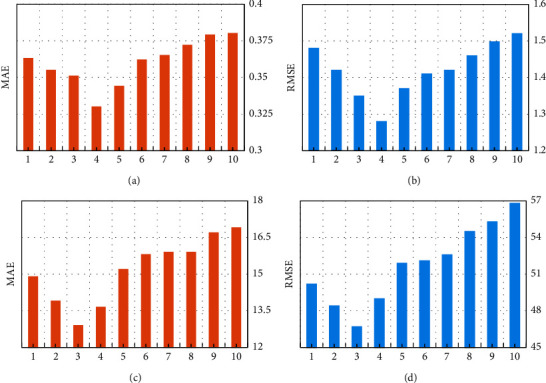
The influence of parameter top-*k*. (a) Top-k on RT attribute. (b) Top-k on RT attribute. (c) Top-k on TP attribute. (d) Top-k on TP attribute.

The parameter *k* can determine how many similar users or similar services of the selected target are to be used. Here, in order to study the influence of top *k*-th values on prediction accuracy, we set the matrix density to 0.05 and *λ* = 0.4, and set the Top-*k* value from 1 to 10 and gradually increase at intervals of 1, the results are shown in Figure 5. Among them, Figures 5(a) and 5(b) are the experimental results in the response time attribute, and Figures 5(c) and 5(d) are the experimental results in the throughput attribute. It can be seen from Figures 5(a) and 5(d) that with increasing the top-*k* value, the prediction accuracy first increases, then decreases, and reaches the maximum when *k* is 4. This shows that the number of similar users or similar services is not always beneficial to improving the prediction accuracy, too few or too many similar users and similar services can both reduce the prediction accuracy.

#### 5.4.4. The Influence of Matrix Density

**Figure 6:**
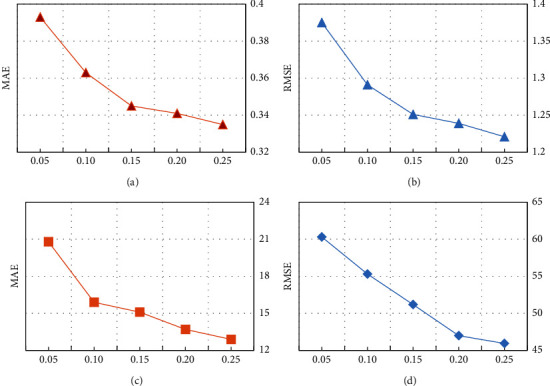
The effect of parameter matrix density. (a) Matrix density on RT. (b) Matrix density on RT. (c) Matrix density on TP. (d) Matrix density on TP.

The matrix density represents what proportion of the QoS matrix is used as training data.  Figure 6 shows the effects of matrix density on two QoS attributes, response time, and throughput, respectively. Here, we set the density of the matrix from 0.05 to 0.25, increasing in 0.05 intervals, *λ* = 0.4, the number of similar users, and the top-*k* is 4.

We can see from Figure 6 that, as the density of the matrix increases, the service prediction accuracy of the RDHE method gradually improves. This is because the larger the matrix density is, the more users have invoked more services, and the generated data set contains more user information and service information, from which more useful information can be mined to improve prediction accuracy.

#### 5.4.5. The Influence of Hyperparameters

Our method includes three neural networks (wide & Deep, GraphSAGE, and MLP for clustering), among which the default values of the parameters have been depicted in Table 1, more discussion can be referred in [39, 40]. Here, we mainly discuss the key parameters of NN, which are crucial for clustering, including the number of hidden layers, learning rate, and dropout.

*(1) Impact of Hidden Layers*. In theory, the multi-hidden layer network structure can learn patterns from the original data, and express the data more abstractly through layer-by-layer feature extraction. Therefore, the more layers, the stronger the expressive ability of the data and the stronger the prediction ability. But this improvement is not without an upper limit, and when the number of hidden layers is too high, the accuracy may drop.

**Figure 7:**
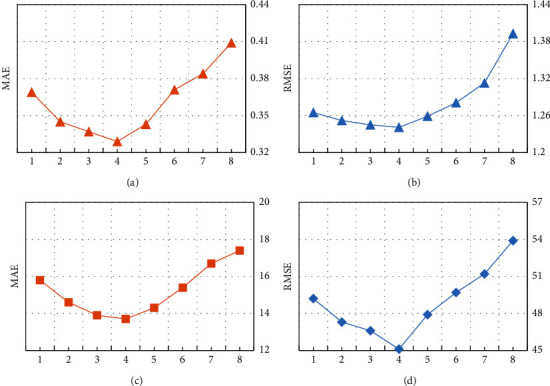
Comparison of the hidden layer numbers. (a) Hidden layer number on RT. (b) Hidden layer number on RT. (c) Hidden layer number on TP. (d) Hidden layer number on TP.

To study the number of impact of the hidden layers, we vary the number of hidden layers from 1 to 8, and the dimension of the hidden layer is set to 64. As shown in Figure 7, in both the RT and TP attributes, when the number of hidden layer increases, the value of MAE also increases. When the number of hidden layers is 4, the optimal prediction effect can be achieved. Therefore, we choose the number of hidden layers as 4 in our method.

**Figure 8:**
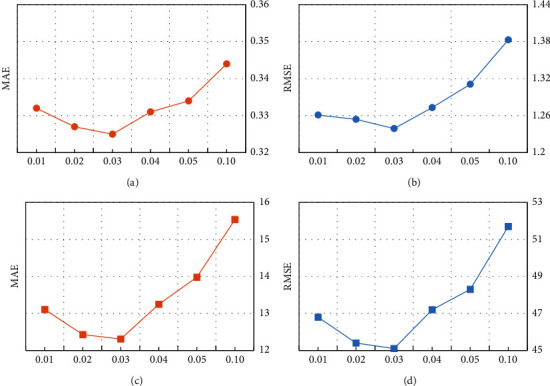
Comparison of the learning rate. (a) Learning rate on RT. (b) Learning rate on RT. (c) Learning rate on TP. (d) Learning rate on TP.

*(2) Impact of Learning Rate*. The learning rate is one of the key hyper-parameters for training neural networks. When the learning rate is relatively small, the loss curve converges very slowly, but the amplitude of the loss swing is relatively small because the amplitude of the weight update amplitude is small. Otherwise, when the learning rate is relatively large, the loss curve converges quickly. Therefore, it is very important to choose the learning rate carefully for our method. In order to find the optimal value of the learning rate, we set the learning rate as 0.01, 0.02, 0.03, 0.04, 0.05, and 0.1.  Figure 8 shows the performance change of RDHE.

From Figures 8(a) and 8(b), we can observe that the learning rate improves the performance when the value range from 0.01 to 0.03 on the RT attribute, and the improvement of the classification effect in the TP attribute does not stop until the learning rate is set to 0.03, this is because a learning rate that is too small can cause the process to get stuck and the method cannot approach the local minima. However, as the value of the learning rate varies from 0.03 to 0.1 on the RT attribute and the TP attribute, the classification performance gradually decreased; the possible cause of this phenomenon is that a learning rate too large will cause the model to converge too quickly to a suboptimal solution. Therefore, in our experiments, the default learning rate is set to 0.03.

*(3) Comparative Results of Node and Feature Dropout*. Although GCN-based learning models have strong representation ability, they are usually affected by overfitting. The dropout strategy is a common method for solving the overfitting of the neural network, which has the characteristics of simplicity and good effect. Here, we employ node and feature dropout strategies to prevent RDHE from overfitting. The feature dropout strategy randomly removes the output information with a probability of *p*_*n*_. As a result, only partial information contributes to the refined representations in the *l*-th propagation layer. We also run a node dropout strategy to randomly block a particular node and discard all its output information with a probability *p*_*f*_.

**Figure 9:**
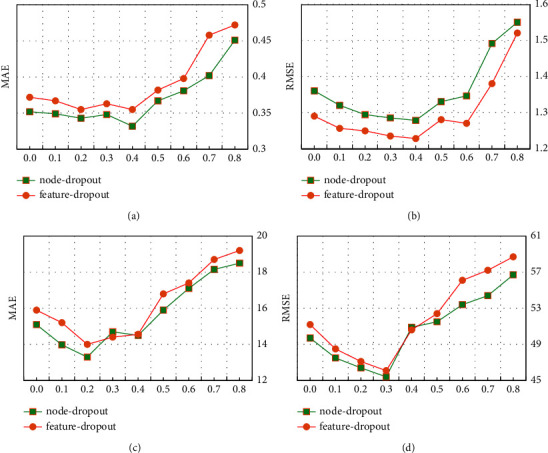
Comparison of dropout ratio. (a) Dropout ratio on RT. (b) Dropout ratio on RT. (c) Dropout ratio on TP. (d) Dropout ratio on TP.

Figure 9 shows the effect of the node dropout ratio and the feature dropout on different attributes of the WS-Dream dataset. From Figure 9, we can find that the node dropout strategy gets better accuracy than feature dropout both on (a), (c), and (d). Taking (a) Dropout Ratio on RT as an example, setting the radio as 0.4 will lead to the highest MAE result of 0.329, which is better than that of the feature dropout result of 0.362. One possible reason is that dropping some features of user or service nodes can make the representation more robust; therefore, node dropout is more effective than feature dropout, which means that the node dropout strategy can be a solution to graph neural network overfitting.

#### 5.4.6. Experimental Analysis of Fast Similarity Calculation Method

In this work, a neural network-based similarity calculation process is designed for downstream similarity calculation and reduced the time complexity. In order to verify the effectiveness of our method, we designed two comparison experiments on TP, (1) FSCM, which had fast similarity calculation component and (2) Non-FSCM, which traversed the embedding one by one directly when calculating sim(*s*_*i*_, *s*_*j*_), the experiment selected five models: LDA, GraphSage, GCN, NGCF, and RDHE for comparison, the unit of Runtime is hours, and the runtime required for the comparison experiment is shown in Table 4.

It can be seen from Table 4 that the addition of the fast similarity calculation component is 4 hours (25.48% less than Non-FSCM) less than the Non-FSCM method on RT and 3.9 hours (24.69% less than Non-FSCM) on TP. It is clear that at the scale of our dataset, the fast similarity calculation method we designed is effective.

## 6. Conclusions and Future Work

This article proposes a novel community discovery and hierarchical embedding method for web service prediction based on a dual representation and similarity calculation mechanism, which can both utilizes attribute information and structural information to achieve a better prediction effect. We also propose a fast similarity calculation method to improve the algorithm's ability to handle large-scale data, which can avoid the curse of dimensionality of traversing all data samples and reduce the time complexity of similarity calculation. A huge amount of experiments had been carried out on the WS-Dream. The experimental results show that compared to the classic methods (NGCF, PinSage, KGAT, GraphRec, and NIA-GCN), the precision of our RDHE method is up to 11.11%, 9.19%, 9.19%, 5.62%, and 3.45% higher.

Although the experiments have verified the effectiveness of the community discovery method introduced in this article, our method is based on the multi-layer representation of Wide &Deep and GraphSage, the architecture is more complex, and it is based on the convenience of label acquisition, but it is actually a difficult task to obtain labels. According to our analysis and cognition, the next improvement directions are: (1) further research on semi-supervised and even unsupervised methods and (2) further research on end-to-end training, without sub-module or staged training in the learning process to directly optimize the tasks.

## Figures and Tables

**Table 1:** Default main hyper parameters setting.

Modules	Main hyper parameters	Value
LDA	Number of topics in LDA	20
	Dirichlet parameter	0.1

Node2vec	Length of Node2vec walk	80
	Number of Node2vec walks	10
	Window size of Node2vec walk	10

GCN	Number of layers	2
	Size of layer convolution layer	64

Wide & deep	Number of layers	2
	Dimensions	128

GraphSAGE	Neighborhood deep neighborhood sample sizes	2
		25

NGCF	Embedding propagation layers	3
	Depth of the NGCF	3
	Top-k	20

PinSage	Top-k	2
	Size of hidden dimension size	512

KGAT	Top-k	20
	Tower structure of hidden layer size	512/256/128/64

GraphRec	Size of the hidden layer	128
	Number of three hidden layers	3

NIA-GCN	Size of first layer/second layer	40/2
	Dimension of each layer	64

**Table 2:** Overall performance comparison of RT on average.

Method	MAE/matrix density						RMSE/matrix density					
	0.10	0.15	0.20	0.25	0.30	Average	0.10	0.15	0.20	0.25	0.30	Average
LDA	0.49	0.46	0.45	0.43	0.42	0.4500	1.48	1.45	1.44	1.43	1.41	1.4420
Node2vec	0.44	0.43	0.41	0.40	0.39	0.4140	1.43	1.41	1.4	1.38	1.37	1.3980
GCN	0.45	0.43	0.43	0.42	0.41	0.4280	1.47	1.45	1.43	1.41	1.39	1.4300
Wide & deep	0.42	0.41	0.39	0.38	0.37	0.3975	1.39	1.36	1.32	1.30	1.28	1.3300
GraphSAGE	0.41	0.4	0.38	0.37	0.36	0.3840	1.38	1.37	1.35	1.34	1.32	1.3520
NGCF	0.4	0.38	0.37	0.38	0.36	0.3780	1.37	1.36	1.35	1.34	1.33	1.3500
PinSage	0.4	0.38	0.36	0.36	0.35	0.3700	1.35	1.34	1.32	1.31	1.30	1.3240
KGAT	0.39	0.38	0.37	0.36	0.35	0.3700	1.31	1.29	1.27	1.26	1.25	1.2760
GraphRec	0.38	0.36	0.35	0.35	0.34	0.3560	1.3	1.27	1.25	1.23	1.22	1.2540
NIA-GCN	0.39	0.36	0.34	0.33	0.32	0.3480	1.29	1.26	1.23	1.22	1.21	1.2420
**RDHE**	**0.37**	**0.35**	**0.34**	**0.32**	**0.3**	**0.3360**	**1.24**	**1.22**	**1.21**	**1.20**	**1.20**	**1.2140**

**Table 3:** Overall performance comparison of TP on average.

Method	MAE/matrix density						RMSE/matrix density					
	0.10	0.15	0.20	0.25	0.30	Average	0.10	0.15	0.20	0.25	0.30	Average
LDA	16.29	15.10	14.02	13.97	13.73	14.6220	58.25	57.2	56.56	55.1	54.55	56.3320
Node2vec	14.85	13.76	13.35	13.19	12.82	13.5940	56.85	55.98	54.15	53.29	51.18	54.2900
GCN	15.25	14.30	14.01	13.83	13.55	14.1880	57.93	57.1	55.97	54.35	53.83	55.8360
Wide & deep	14.98	14.21	14.02	13.87	13.85	14.1860	56.09	55.12	54.4	52.97	51.95	54.1060
GraphSAGE	14.76	14.10	13.66	13.11	12.97	13.7200	55.57	54.04	53.52	52.81	53.19	53.8260
NGCF	14.85	14.20	13.86	13.31	12.85	13.8140	55.06	54.02	53.21	51.56	50.94	52.9580
PinSage	14.69	14.15	13.78	13.23	12.95	13.7600	52.87	51.83	50.3	49.78	48.96	50.7480
KGAT	14.76	14.10	13.66	13.11	12.97	13.7200	51.87	51.33	50.25	49.98	48.85	50.4560
GraphRec	14.18	13.53	12.95	12.86	12.75	13.2540	54.23	53.6	52.15	51.42	50.07	52.2940
NIA-GCN	13.58	13.22	13.05	12.99	12.87	13.1420	52.23	51.73	50.05	49.68	48.77	50.4920
**RDHE**	**12.58**	**12.49**	**12.45**	**12.40**	**12.17**	**12.4180**	**48.26**	**47.41**	**46.16**	**45.32**	**45.01**	**46.4300**

**Table 4:** Runtime comparison of TP on average (hour).

Models	RT		TP	
	FSCM (h)	Non-FSCM (h)	FSCM (h)	Non-FSCM (h)
LDA	7.5	10.7	7.6	10.9
GraphSage	8.9	14.3	9.1	14.6
GCN	12.1	16.5	12.5	15.9
NGCF	14.7	18.2	14.9	18.4
RDHE	15.2	18.4	15.4	18.6
**Average**	**11.7**	**15.7**	**11.9**	**15.8**

## Data Availability

The WS-Dream dataset can be obtained at https://github.com/wsdream/wsdream-dataset.
